# Separation of New Coumarin Glycosides from *Toddalia asiatica* Using Offline Two-Dimensional High-Performance Liquid Chromatography

**DOI:** 10.3390/plants9040428

**Published:** 2020-03-31

**Authors:** Yan Li, Shi-Wei Sun, Xiao-Yi Zhang, Yang Liu, Xiao-Hong Liu, Shuang Zhang, Wei Wang, Jin Wang, Wei Wang

**Affiliations:** 1 Department of Natural Medicine and Pharmacognosy, School of Pharmacy, Qingdao University, Qingdao 266071, China; liyanyaohua@126.com (Y.L.); sunsw@qdu.edu.cn (S.-W.S.); buckuper@163.com (Y.L.); liuxiaohong1043@163.com (X.-H.L.); qdeduzhangshuang@163.com (S.Z.); justwangwade@126.com (W.W.); Qingdao_wangjin@163.com (J.W.); 2 School of Pharmacy, Jilin University, Changchun 130021, China; Zhangxy925814926@163.com

**Keywords:** coumarin glycosides, *Toddalia asiatica*, two-dimensional high-performance liquid chromatography

## Abstract

Coumarins and flavonoids are the major constituents of *Toddalia asiatica*. The separation and purification of ingredients from *T*. *asiatica* is an important procedure to acquire high-purity compounds for subsequent pharmacological investigation to discover leading compounds. In the present work, an offline two-dimensional high-performance liquid chromatography (HPLC) method was successfully established for the separation of high-purity glycosides from *T. asiatica*. Based on the separation results obtained with two different chromatographic stationary phases, a phenyl-bonded silica-based reversed-phase column was employed as the first HPLC preparation, and three fractions were obtained from the sample. Then, the fractions were isolated and purified on an octadecyl-bonded silica-based reversed-phase column to obtain high-purity compounds in the second HPLC separation. As a result, three coumarin glycosides, including two undescribed and one known, along with one known flavonoid glycoside with more than 98% purity were isolated from the sample. The structures of the isolated compounds were elucidated on the basis of extensive spectroscopic evidence derived from optical rotation, mass spectrometry, and nuclear magnetic resonance experiments. Two-dimensional HPLC with different stationary phases has the potential to be an efficient method for the separation of high-purity compounds from *T*. *asiatica*.

## 1. Introduction

*Toddalia asiatica* (L.) Lam. (Rutaceae) (Syn: *Toddalia aculeata* Pers., *Scopolia aculeata* Sm., *Paullinia asiatica* L.), well known as Feilongzhangxue in Chinese and commonly known as orange climber in English, is widely distributed in the east, south, and southeast of Asia, Madagascar, Africa, and the Mascarene Islands [1,2]. The roots and barks of *T*. *asiatica* have been used in Miao minority medicine, mainly in Guizhou, Yunnan, and Guangxi provinces in China, for the treatment of fall injuries, rheumatic arthralgia, stomachache, chronic lumbago, and diarrhea [3,4]. Previous phytochemical and pharmacological investigations of different parts of this plant revealed that coumarins are the main secondary metabolites [5], some of which exhibited cytotoxic, antimalarial [6], antiviral [7], anti-inflammatory [8], antibacterial [9], anti-platelet aggregation [10], anti-plasmodial, larvicidal [11], phosphodiesterase-4 inhibitory [12], and osteoclastogenesis activities [13]. Even though coumarins are rather studied, less attention has been paid to their glycosides. As far as we know, coumarin glycosides tend to be used for the treatment and prevention of diseases. For example, Semelil (Angipars™), a *Melilotus officinalis* (L.) Lam. extract mainly containing coumarin glycosides, may assist in the management of wounds and varicose veins [14,15]. Thus, it is desirable to acquire high-purity coumarin glycosides from *T*. *asiatica* for subsequent pharmacological investigation to discover leading compounds.

To date, the isolation of compounds from *T. asiatica* have mainly relied on traditional column chromatography over a silica-based normal phase, an octadecyl-bonded silica-based reversed-phase, and sephadex LH-20. One-dimensional high-performance liquid chromatography (HPLC) was widely applied for the purification of single compounds in complex fractions. Most of one-dimensional HPLC-based isolations of compounds from *T. asiatica* were carried out with an octadecyl-bonded silica-based reversed-phase stationary phase because of its universality [6,7,8,9,10,11,12,13]. However, during the course of our search for coumarin glycosides, due to their similar structure and limited chromatographic resolution, it was difficult to directly obtain high-purity compounds using only one-dimensional HPLC techniques. In recent years, different two-dimensional HPLC methods have been proven to be more comprehensive and effective natural product isolation techniques, since they are based on different separation mechanisms and designed to improve separation selectivity [16]. Selecting appropriate stationary phases might be of great importance to ensure that different compounds can be selectively isolated. The most common stationary phase is the octadecyl-bonded silica-based stationary phase. Recently, phenyl-bonded silica-based stationary phases have been theorized to provide additional selectivity for the separation of natural products, due to their capability to promote π–π interactions between the electron-rich phenyl ring and a solute, which modifies the retention mechanism and contributes to the separation of compounds [17]. Jiang et al. indicated that the phenyl-bonded stationary phase could regulate various interactions by varying the substituents (electron-withdrawing or electron-donating) on benzene, leading to an extended application in the separation of phenolic compounds [18]. For example, Janas et al. reported the separation of 11 various flavonoids on different phenyl-bonded stationary phases in reversed-phase conditions [19]. Thus, the phenyl-bonded silica-based stationary phase could compensate for the polar selectivity of the octadecyl-bonded silica-based stationary phase during the separation procedure.

In this study, using two reversed-phase columns with phenyl- and octadecyl-bonded silica-based stationary phases, we developed a two-dimensional HPLC method to realize a high-performance separation of coumarin and flavonoid glycosides from the roots of *T. asiatica* and obtain high-purity compounds. To our knowledge, this is the first report on separating glycosides from *T. asiatica* using two-dimensional HPLC. This method could be a useeful tool for the separation of high-purity compounds from *T. asiatica*, which will facilitate the investigation of their pharmacological activities.

## 2. Results and Discussion

### 2.1. Construction of a Two-Dimensional HPLC System

**Figure 1:**
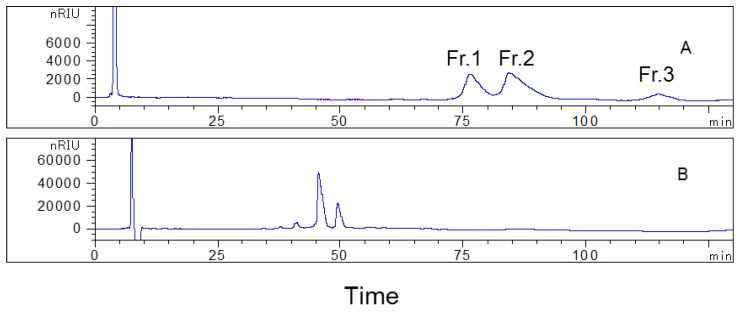
HPLC preparation chromatograms of the examined sample using the Senshu Pak C_6_H_5_-3152-N column (150 × 8 mm, **A**) and the YMC-Pack ODS-AQ column (250 × 10 mm, **B**). Conditions: mobile phase: water and methanol (80:20, *v*/*v*) and water–acetonitrile (82:18, *v*/*v*) for (**A**) and (**B**), respectively; flow rates: 1.5 mL/min and 2.0 mL/min for (**A**) and (**B**), respectively; column temperature: 30 °C.

During the course of our search for coumarin glycosides, a fraction eluted with 60% methanol was isolated by one-dimensional HPLC using an octadecyl-bonded silica-based reversed-phase YMC-Pack ODS-AQ column and water–acetonitrile (82:18, *v*/*v*) as the mobile phase (Figure 1). However, we could not obtain high-purity compounds directly. In order to construct a two-dimensional HPLC method to realize efficient separation of high-purity compounds, a phenyl-bonded silica-based reversed-phase Senshu Pak C_6_H_5_-3152-N column was tested. As shown in Figure 1, different chromatographic results were obtained with the Senshu Pak C_6_H_5_-3152-N column with respect to those previously obtained. Different separation patterns were observed when using these two columns, which indicated that they had different selectivity for the separation of compounds present in the sample. The Senshu Pak C_6_H_5_-3152-N and YMC-Pack ODS-AQ columns were both polar, copolymerized, reversed-phase columns, according to the manufacturer. However, compared with the octadecyl-bonded silica-based stationary phase, the phenyl-bonded silica-based stationary phase has unique separation properties. Its selectivity towards aromatic compounds is different from that of the octadecyl-bonded silica gel stationary phase, which is caused by the interactions occurring between π electrons in the aromatic moiety and the solute molecules [17,20]. Therefore, we speculated that a two-dimensional HPLC method combining these two columns might be highly selective for the separation of high-purity coumarin glycosides. The Senshu Pak C_6_H_5_-3152-N column was utilized in the first HPLC separation to obtain several fractions from the sample. Subsequently, in the second HPLC separation, the YMC-Pack ODS-AQ column was used to obtain compounds with high purity, as a result of its different selectivity compared with the phenyl-bonded silica-based stationary phase.

### 2.2. First HPLC Separation

The optimized conditions were established by adjusting the composition of mobile phase, flow rate, and injection volume. The first HPLC separation was performed on a phenyl-bonded silica-based reversed-phase Senshu Pak C_6_H_5_-3152-N column with the mobile phase consisting of water and methanol (80:20, *v*/*v*), at a constant flow rate of 1.5 mL/min. The sample loading was 0.02 g, and the time of each separation was 125 min (Figure 1A). Fractions with obvious peaks were separated and collected (Fr. 1, 75–80 min; Fr. 2, 84–92 min; and Fr. 3, 110–120 min), and the same fractions of each preparation were combined to enrich the target compounds. In total, three fractions were collected in the first HPLC separation.

### 2.3. Analysis of the Collected Fractions on the YMC-Pack ODS-AQ Column

**Figure 2:**
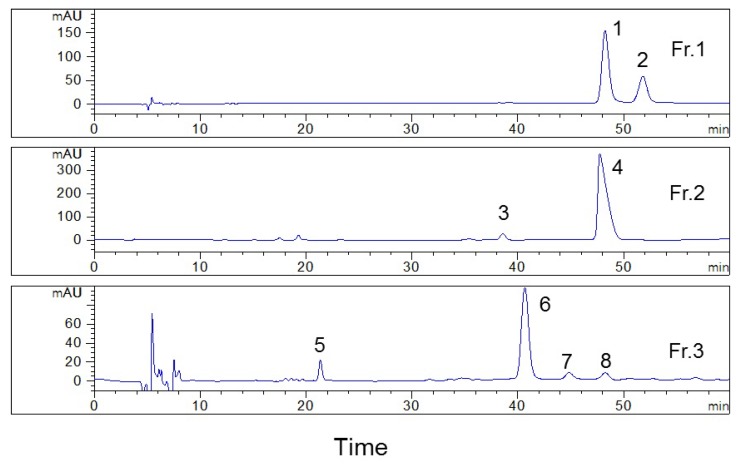
Analytical HPLC chromatograms of fractions collected during the first HPLC separation on the YMC-Pack ODS-AQ column (250 × 4.6 mm). Conditions: mobile phase: water–acetonitrile (82:18, *v*/*v*); flow rate: 0.42 mL/min; column temperature: 25 °C; monitoring wavelength: 269 nm.

Fractions 1, 2, and 3 were analyzed on the YMC-Pack ODS-AQ column (Figure 2) to compare the separation selectivity between the Senshu Pak C_6_H_5_-3152-N column and the YMC-Pack ODS-AQ column. The results showed that these two columns have good orthogonality. The first HPLC separation efficiently simplified the sample and improved the separation on the second HPLC, so that multiple compounds with high purity could be prepared using the two-dimension HPLC method. On one hand, peaks 1, 4, and 8 had similar retention times on the YMC-Pack ODS-AQ column, and the first HPLC separation on Senshu Pak C_6_H_5_-3152-N column mainly eluted peak 1 into fraction 1, peak 4 into fraction 2, and peak 8 into fraction 3. These three peaks could be isolated from three fractions, so to minimize interference between each other. If the sample was applied on the YMC-Pack ODS-AQ column directly, these three peaks were difficult to be separated. These peaks could not be purified without the first HPLC separation. On the other hand, peaks 1 and 2 in fraction 1 had the same retention time on the Senshu Pak C_6_H_5_-3152-N column but were satisfactorily resolved on the YMC-Pack ODS-AQ column. Thus, the compounds characterized by these two peaks could be easily prepared with high purity in the second HPLC. However, for fractions 2 and 3, despite the fact that they showed single peaks on the Senshu Pak C_6_H_5_-3152-N column, other impurities, indicated in the Figure as 3, 5, 7, and 8, were present in the isolate when the YMC-Pack ODS-AQ column was used. 

### 2.4. Second HPLC Preparation of High-Purity Compounds

**Figure 3:**
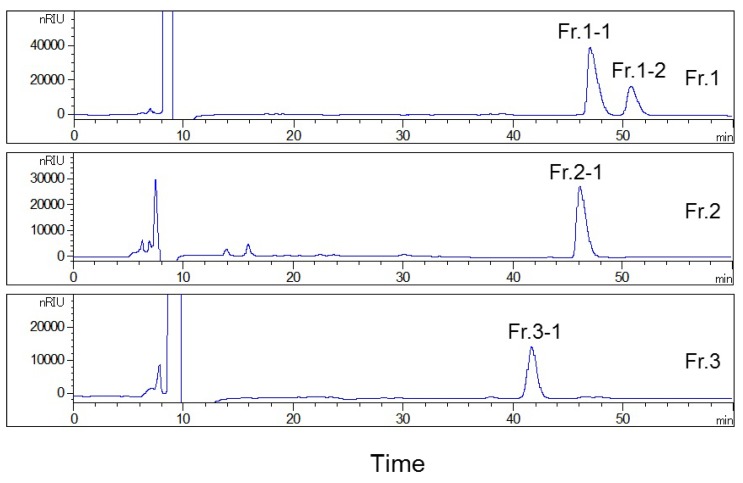
HPLC preparation chromatograms of fractions collected during the first HPLC separation on the YMC-Pack ODS-AQ column (250 × 10 mm). Conditions: mobile phase: water–acetonitrile (82:18, *v*/*v*); flow rate: 2.0 mL/min; column temperature: 30 °C.

**Figure 4:**
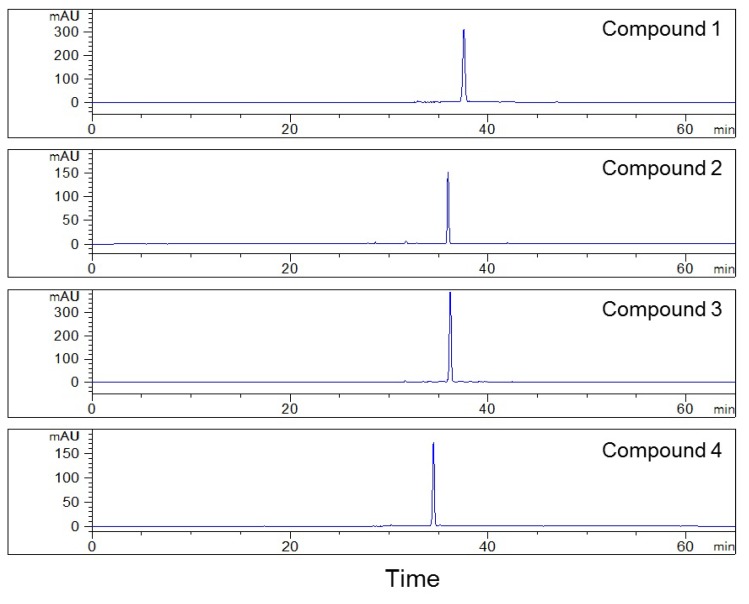
Purity evaluations of the separated compounds on the YMC-Pack ODS-AQ column (250 × 4.6 mm). Conditions: mobile phase: A: 0.2% formic acid in water, and B: acetonitrile; gradient: 0–50 min, 10%–30% B, 50–65 min, 30% B; flow rate: 1.0 mL/min; column temperature: 25 °C; monitoring wavelength: 269 nm.

**Figure 5:**
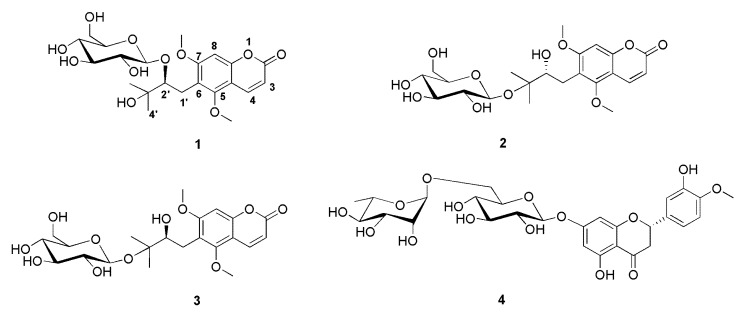
Chemical structures of the separated compounds **1**–**4**.

Based on these results, all fractions were purified in the second HPLC using the YMC-Pack ODS-AQ column to improve their purity, for fraction 1 presented two distinct peaks, while the purity of fractions 2 and 3 was less than 85% after the first HPLC separation (Figure 2). The separation conditions, including the composition of mobile phase and flow rate, were optimized on an analytical YMC-Pack ODS-AQ column and were then used for the separation using the preparative column after conversion. Finally, the isocratic condition was adopted, using water and acetonitrile (82:18, *v*/*v*) as the mobile phase to separate the fractions collected during the first HPLC separation, at a constant flow rate 2.0 mL/min (Figure 3). After isolating fractions 1, 2, and 3, four compounds with purity >98% were obtained, as shown in Figure 4. The structures of the isolated compounds **1** (Fr. 1–2, peak 2), **2** (Fr. 2–1, peak 4), **3** (Fr.1–1, peak 1), and **4** (Fr. 3–1, peak 6) were elucidated on the basis of extensive spectroscopic evidence derived from optical rotation, mass spectrometry, and nuclear magnetic resonance experiments and by comparing their spectroscopic data with those found in the literature. In all, three coumarin glycosides (**1**–**3)**, including (−)-toddalolactone 2′-*O*-*β*-d-glucopyranoside (**1**) and a pair of enantiomers of toddalolactone 3′-*O*-*β*-d-glucopyranoside (**2** and **3**) [12], along with one known flavonoid glycoside hesperidin (**4**) [21] were characterized. The chemical structures of the prepared compounds are shown in Figure 5. The results show that the compounds could be well separated and purified by the two-dimensional HPLC method with phenyl- and octadecyl-bonded silica-based reversed-phases. These two columns had complementary selectivity, and minor compounds could also be enriched by repeated collection.

### 2.5. Structure Identification

**Figure 6:**
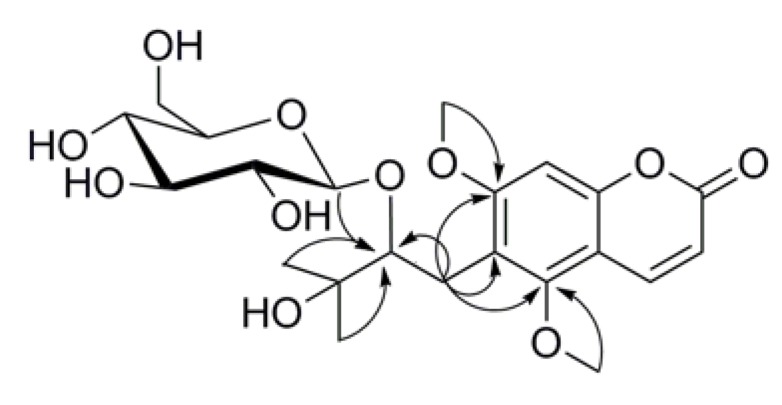
Key HMBC (H→C) correlations for compound **1**.

Compound **1** was obtained as a colorless oil with a negative optical rotation ([*α*]_D_^25^ -41). The molecular formula of **1** was found to be C_22_H_30_O_11_ by the HR-ESIMS ion at m/z 493.1679 [M+Na]^+^ (calculated for C_22_H_30_O_11_Na 493.1680), which was further supported by its ^1^H and ^13^C NMR data. The ^1^H NMR spectrum exhibited the characteristic AB system signals of the lactone ring at *δ* 6.23 (1H, d, *J* = 9.6 Hz, H-3) and 8.02 (1H, d, *J* = 9.6 Hz, H-4) and a singlet signal at *δ* 6.75 (1H, s, H-8), indicative of the presence of a 5,6,7-triisubstituted coumarin moiety. The characteristic signal of the hydroxylated isopentyl group was observed at *δ*_H_ 2.73 (1H, dd, *J* = 13.8, 2.9 Hz, H-1′a), 3.01 (1H, m, H-1′b), 3.89 (1H, dd, *J* = 10.1, 2.9 Hz, H-2′), 1.31 (3H, s, H-4′), and 1.26 (3H, s, H-5′) and *δ*_C_ 26.9 (C-1′), 88.0 (C-2′), 75.0 (C-3′), 26.4 (C-4′), and 24.1 (C-5′), following the HSQC, ^1^H-^1^H COSY, and HMBC correlations (Figure 6). The substituted position was determined by the HMBC correlations from H-1′ to C-6 (*δ*_C_ 119.8) of the coumarin moiety. A two-methoxy-group-substituted coumarin moiety was confirmed by the presence of the methoxy groups resonating at *δ*_H_ 3.92 (3H, s) and 3.93 (3H, s) in the ^1^H NMR spectrum and *δ*_C_ 56.8 and 64.1 in the ^13^C NMR spectrum, along with the HMBC correlations from the methoxy hydrogens resonating at *δ*_H_ 3.92 and 3.93 to C-5 (*δ*_C_ 157.8) and C-7 (*δ*_C_ 163.9) of the coumarin moiety, respectively. The ^13^C NMR spectrum displayed the existence of 22 carbons, including 9 coumarin carbons, 2 methoxy carbons, and a set of isopentyl group carbons. The remaining six oxygenated carbons were assignable to one sugar unit. By comparison of the NMR data with those reported [12], the hexose was determined as a glucopyranosyl moiety [*δ*_H_ 4.19 (1H, d, J = 7.7 Hz, H-1″) and *δ*_C_ 106.0 (C-1″), 75.9 (C-2″), 78.0 (C-3″), 71.6 (C-4″), 77.1 (C-5″), and 62.8 (C-6″)]. The aforementioned data indicated that the chemical structure of **1** resembled that of toddalolactone 3′-*O*-*β*-d-glucopyranoside (compound **3**) [12], except for the chemical shift of C-2′ in compound **1** to a downfield (*δ*_C_ 88.0 in compound **1**, *δ*_C_ 77.5 in compound **3**), whereas the chemical shift of C-3′ in compound **1** shifted to an upfield (*δ*_C_ 75.0 in compound **1,** *δ*_C_ 81.9 in compound **3**), which indicated that the *β*-d-glucopyranosyl moiety in compound **1** was located at the C-2′ position. The above speculation was confirmed by HMBC correlations from *δ*_H_ 4.19 to *δ*_C_ 88.0. Meanwhile, the optical rotation of this compound was [*α*]_D_^25^ -41 (*c* 0.2, CH_3_OH), which was consistent with that of compound **3** [*α*]_D_^25^ -44 (*c* 0.2, CH_3_OH). On the basis of the abovementioned evidence, the structure of compound **1** was determined as 6-[(2′S)-2′-*O*-*β*-d-glucopyranosyloxy-3′-hydroxy-3′-methylbutyl]-5,7-dimethoxy-2H-1-benzopyran-2-one, and the compound was given the trivial name of (−)-toddalolactone 2′-*O*-*β*-d-glucopyranoside (Figures S1–S6).

Compound **2** exhibited the molecular formula C_22_H_30_O_11_, as determined by 1D NMR data and the HRESIMS ion at *m*/*z* 471.1862 [M+H]^+^ (calcd. for C_22_H_31_O_11_, 471.1861). Its ^1^H as well as the ^13^C NMR data demonstrated that compound **2** possessed 22 carbons displaying coumarin glycoside features closely similar to those of (−)-toddalolactone 3′-*O*-*β*-d-glucopyranoside (**3**) [12]. However, compounds **2** and **3** exhibited two well-resolved peaks with different retention times on the phenyl-bonded silica-based reversed-phase Senshu Pak C_6_H_5_-3152-N column, indicating **2** may be the enantiomer of **3**. This was supported by the optical rotation of **2** [*α*]_D_^25^ +44 (*c* 0.2, CH_3_OH), which was opposite to that of **3** [*α*]_D_^25^ -44 (*c* 0.2, CH_3_OH). Thus, **2** was identified as the enantiomer of **3** and given the trivial name (+)-toddalolactone-3′-*O*-*β*-d-glucopyranoside (Figures S7–S12).

Compound **1**: colorless oil; [*α*]_D_^25^ -41 (*c* 0.2, CH_3_OH). ^1^H NMR (CD_3_OD, 500 MHz) *δ* 8.02 (1H, d, *J* = 9.6 Hz, H-4), 6.75 (1H, s, H-8), 6.23 (1H, d, *J* = 9.6 Hz, H-3), 4.19 (1H, d, *J* = 7.7 Hz, H-1″), 3.93 (3H, s, 7-OCH_3_), 3.92 (3H, s, 5-OCH_3_), 3.89 (1H, dd, *J* = 10.1, 2.9 Hz, H-2′), 3.27 (1H, dd, *J* = 11.3, 3.0 Hz, H-6″b), 3.21 (1H, m, H-3″), 3.19 (1H, m, H-6″a), 3.06 (1H, m, H-2″), 3.05 (1H, m, H-4″), 3.01 (1H, m, H-1′b), 2.87 (1H, m, H-5″), 2.73 (1H, dd, *J* = 13.8, 2.9 Hz, H-1′a), 1.31 (3H, s, H-4′), 1.26 (3H, s, H-5′); ^13^C NMR (CD_3_OD, 125 MHz) *δ* 163.9 (C-7), 163.5 (C-2), 157.8 (C-5), 156.2 (C-9), 141.3 (C-4), 119.8 (C-6), 112.4 (C-3), 108.4 (C-10), 106.0 (C-1″), 96.2 (C-8), 88.0 (C-2′), 78.0 (C-3″), 77.1 (C-5″), 75.9 (C-2″), 75.0 (C-3′), 71.6 (C-4″), 64.1 (5-OCH_3_), 62.8 (C-6″), 56.8 (7-OCH_3_), 26.9 (C-1′), 26.4 (C-4′), 24.1 (C-5′); HRESIMS m/z 493.1679 [M+Na]+ (calcd for C_22_H_31_O_11_Na, 493.1680).

Compound **2**: colorless oil; [*α*]_D_^25^ +44 (*c* 0.2, CH_3_OH), ^1^H NMR(CD_3_OD, 500 MHz) δ 8.02 (1H, d, *J* = 9.6 Hz, H-4), 6.74 (1H, s, H-8), 6.23 (1H, d, *J* = 9.6 Hz, H-3), 4.56 (1H, d, J = 7.7 Hz, H-1″), 3.91 (3H, s, 7-OCH_3_), 3.90 (3H, s, 5-OCH_3_), 3.82 (1H, m, H-6″b), 3.80 (1H, t, H-2′), 3.62 (1H, dd, *J* = 11.7, 5.0 Hz, H-6″a), 3.37 (1H, m, H-3″), 3.31 (1H, m, H-5″), 3.29 (1H, m, H-4″), 3.19 (1H, m, H-2″), 2.88 (1H, m, H-1′b), 2.76 (1H, dd, *J* = 13.6, 2.5 Hz, H-1′a), 1.36 (3H, s, H-5′), 1.36 (3H, s, H-4′); ^13^C NMR (CD_3_OD, 125 MHz) δ 163.7 (C-7), 163.3 (C-2), 157.7 (C-5), 156.2 (C-9), 141.2 (C-4), 120.2 (C-6), 112.6 (C-3), 108.4 (C-10), 98.6 (C-1″), 96.3 (C-8), 81.9 (C-3′), 78.1 (C-3″), 77.7 (C-5″), 77.5 (C-2′), 75.2 (C-2″), 71.7 (C-4″), 63.9 (5-OCH_3_), 62.7 (C-6″), 56.7 (7-OCH_3_), 27.1 (C-1′), 23.9 (C-5′), 21.8 (C-4′); HRESIMS 471.1862 [M+H]^+^ (calcd for C_22_H_31_O_11_, 471.1861).

## 3. Materials and Methods

### 3.1. Apparatus and Reagents

HPLC separation was carried out on a system consisting of a Prepstar SD-1 solvent delivery unit, a G1316A thermostatted column compartment, a G1362A refractive index detector, and an Agilent HPLC workstation (Agilent technologies, Santa Clara, CA, USA). HPLC analysis was performed on an Agilent 1260 system equipped with a G1311C quaternary pump, a G1314F variable-wavelength detector coupled with an analytical workstation, a G1329B autosampler, and a G1316A thermostatted column compartment. The columns used in this study were: phenyl-bonded silica-based reversed-phase Senshu Pak C_6_H_5_-3152-N column (150 × 8 mm, i.d., 5 µm, Senshu Scientific, Tokyo, Japan), octadecyl-bonded silica-based reversed-phase YMC-Pack ODS-AQ column (250 × 10 mm, i.d., 5 μm), and YMC-Pack ODS-AQ column (250 × 4.6 mm, i.d., 5 μm, YMC, Kyoto, Japan).

Mass spectra were obtained using an LTQ Orbitrap XL mass spectrometer (Thermo Fisher Scientific, Waltham, MA, USA). A JASCO P-1020 automatic digital polarimeter (JASCO, Tokyo, Japan) was used for the optical rotation experiments. Nuclear magnetic resonance spectra were measured on a Bruker AV-500 FT-NMR spectrometer (Bruker Daltonics, Bremen, Germany) in deuterated methanol, using visual CD_3_OD resonances (^1^H *δ* 3.31 and ^13^C *δ* 49.0) as internal reference. 

Chromatographic-grade acetonitrile and methanol were bought from Oceanpak (Goteborg, Sweden). Ethyl acetate, *n*-butanol, ethanol, methanol, and formic acid purchased from Fuyu Fine Chemical (Tianjin, China) were of analytical grade. Deionized water was purified using a Milli-Q water purification system (Millopore, Bedford, MA, USA). All samples and reagents prepared for chromatographic analysis and separation were filtered through 0.45 μm membranes (Jinteng Experimental Equipment, Tianjin, China) before use.

The air-dried roots of *T. asiatica* were purchased from Jingde Pharmaceutical Company, Bozhou, Anhui Province of China, in October 2017, and authenticated by Prof. Yingxia Li, School of Pharmacy, Qingdao University, China. A voucher specimen (accession number: TA20171001) was deposited at the School of Pharmacy, Qingdao University, China.

### 3.2. Sample Preparation

The raw materials (11.80 kg) were powdered and reflux-extracted with 90% ethanol for 2 h at 80 °C. After evaporating the solvent under reduced pressure, the residue (1.56 kg) was dissolved in distilled water (2 L) and partitioned with ethyl acetate (2 L × 3) and *n*-butanol (2 L × 3), successively, and then concentrated to yield 596.4 g of the ethyl acetate extract and 241.2 g of the *n*-butanol extract. The *n*-butanol fraction (8 g) was chromatographed on an octadecyl-bonded silica-based reversed-phase column and eluted with methanol–water (30:70 and 60:40), successively. The fraction eluted with 60% methanol (1.2 g) was chosen for subsequent two-dimensional HPLC separation. The target sample was dissolved in 6 mL of methanol and stored at 4 °C until further separation.

### 3.3. Chromatographic Conditions

The first HPLC isolation of the fraction eluted with 60% methanol was carried out on a phenyl-bonded silica-based reversed-phase Senshu Pak C_6_H_5_-3152-N column (150 × 8 mm). The mobile phase consisted of water and methanol (80:20, *v*/*v*) at a constant flow rate of 1.5 mL/min. The column temperature was maintained at 30 °C, and the chromatogram was recorded using a refractive index detector. The sample injection volume was 100 μL.

The second HPLC preparation was performed on a YMC-Pack ODS-AQ (250 × 10 mm) column. The mobile phase of water and acetonitrile (82:18, *v*/*v*) was adopted to separate the fractions collected during the first HPLC separation at a constant flow rate of 2.0 mL/min. The column temperature was maintained at 30 °C, and the chromatogram was recorded using a refractive index detector. The sample injection volume was 200 μL.

The HPLC analysis of the fractions collected during the first HPLC separation was carried out on a YMC-Pack ODS-AQ column (250 × 4.6 mm) at 25 °C. The mobile phase was the same as that of the second HPLC preparation, and the flow rate was 0.42 mL/min. The detection wavelength was 269 nm, and the sample injection volume was set at 10 μL.

The HPLC analysis of the compounds purified from the three fractions was performed on a YMC-Pack ODS-AQ column (250 × 4.6 mm) at 25 °C. The mobile phase consisted of water with 0.2% formic acid (A) and acetonitrile (B). The gradient elution program was as follows: 0–50 min, 10–30% B, 50–65 min, 30% B at a constant flow rate of 1.0 mL/min. The detection wavelength was 269 nm, and the sample injection volume was set at 10 μL.

## 4. Conclusions

An offline two-dimensional HPLC system based on two reversed-phase columns with phenyl- and octadecyl-bonded silica-based stationary phases was used for the isolation of the target compounds from *T. asiatica* at high-purity. The Senshu Pak C_6_H_5_-3152-N column was used to simplify the sample in the first HPLC separation. Then, the YMC-Pack ODS-AQ column was adopted to isolate and purify compounds in the second HPLC preparation. Benefiting from the optimized collection mode, four glycosides with purity higher than 98% were obtained in the second HPLC separation. Among them, (−)-toddalolactone 2′-*O*-*β*-d-glucopyranoside and (+)-toddalolactone-3′-*O*-*β*-d-glucopyranoside are new coumarin glycosides. The methodology used in this investigation based on two different chromatographic stationary phases with different separation mechanisms allowed obtaining the target compounds in a satisfactory degree of purity, which will facilitate the investigation on their pharmacological activities.

## Supplementary Materials

The following are available online at https://www.mdpi.com/2223-7747/9/4/428/s1, Figure S1: HRESIMS spectrum of compound **1**, Figure S2: 1H NMR spectrum of compound **1**, Figure S3: 13C NMR spectrum of compound **1**, Figure S4: 1H-1H COSY spectrum of compound **1**, Figure S5: HSQC spectrum of compound **1**, Figure S6: HMBC spectrum of compound **1**, Figure S7: HRESIMS spectrum of compound **2**, Figure S8: 1H NMR spectrum of compound **2**, Figure S9: 13C NMR spectrum of compound **2**, Figure S10: 1H-1H COSY spectrum of compound **2**, Figure S11: HSQC spectrum of compound **2**, Figure S12: HMBC spectrum of compound **2**.
