# Supplementary material for: Separation of New Coumarin Glycosides from Toddalia asiatica Using Offline Two-Dimensional High-Performance Liquid Chromatography
Source: Plants (Basel). 2020 Mar 31;9(4):428. doi: 10.3390/plants9040428 (PMC7238425; doi:10.3390/plants9040428)
Supplement: Supplementary file 1 [file plants-09-00428-s001.zip › Supplementary Materials.docx]

Supplementary Materials

Separation of New Coumarin Glycosides from *Toddalia asiatica* Using Offline Two-Dimensional High-Performance Liquid Chromatography

Yan Li ^1,†^, Shi-Wei Sun ^1,†^, Xiao-Yi Zhang ^2^, Yang Liu ^1^, Xiao-Hong Liu ^1^, Shuang Zhang ^1^, Wei Wang ^1^, Jin Wang ^1^ and Wei Wang ^1,^*

^1^ Department of Natural Medicine and Pharmacognosy, School of Pharmacy, Qingdao University, Qingdao 266071, China; liyanyaohua@126.com (Y.L.); sunsw@qdu.edu.cn (S.-W.S.); buckuper@163.com (Y.L.); liuxiaohong1043@163.com (X.-H.L.); qdeduzhangshuang@163.com (S.Z.); justwangwade@126.com (W.W.); Qingdao_wangjin@163.com (J.W.)

^2^ School of Pharmacy, Jilin University, Changchun 130021, China; Zhangxy925814926@163.com

***** Correspondence: w.w.wangwei@263.net; Tel.: +86-532-8699-1172

^†^ These authors contribute equally to this work and joint first authors.

Supporting information

Figure S1. HRESIMS spectrum of compound 1

Figure S2. ^1^H NMR spectrum of compound 1

Figure S3. ^13^C NMR spectrum of compound 1

Figure S4. ^1^H-^1^H COSY spectrum of compound 1

Figure S5. HSQC spectrum of compound 1

Figure S6. HMBC spectrum of compound 1

Figure S7. HRESIMS spectrum of compound 2

Figure S8. ^1^H NMR spectrum of compound 2

Figure S9. ^13^C NMR spectrum of compound 2

Figure S10. ^1^H-^1^H COSY spectrum of compound 2

Figure S11. HSQC spectrum of compound 2

Figure S12. HMBC spectrum of compound 2

|  |
| --- |

Figure S1. HRESIMS spectrum of compound 1


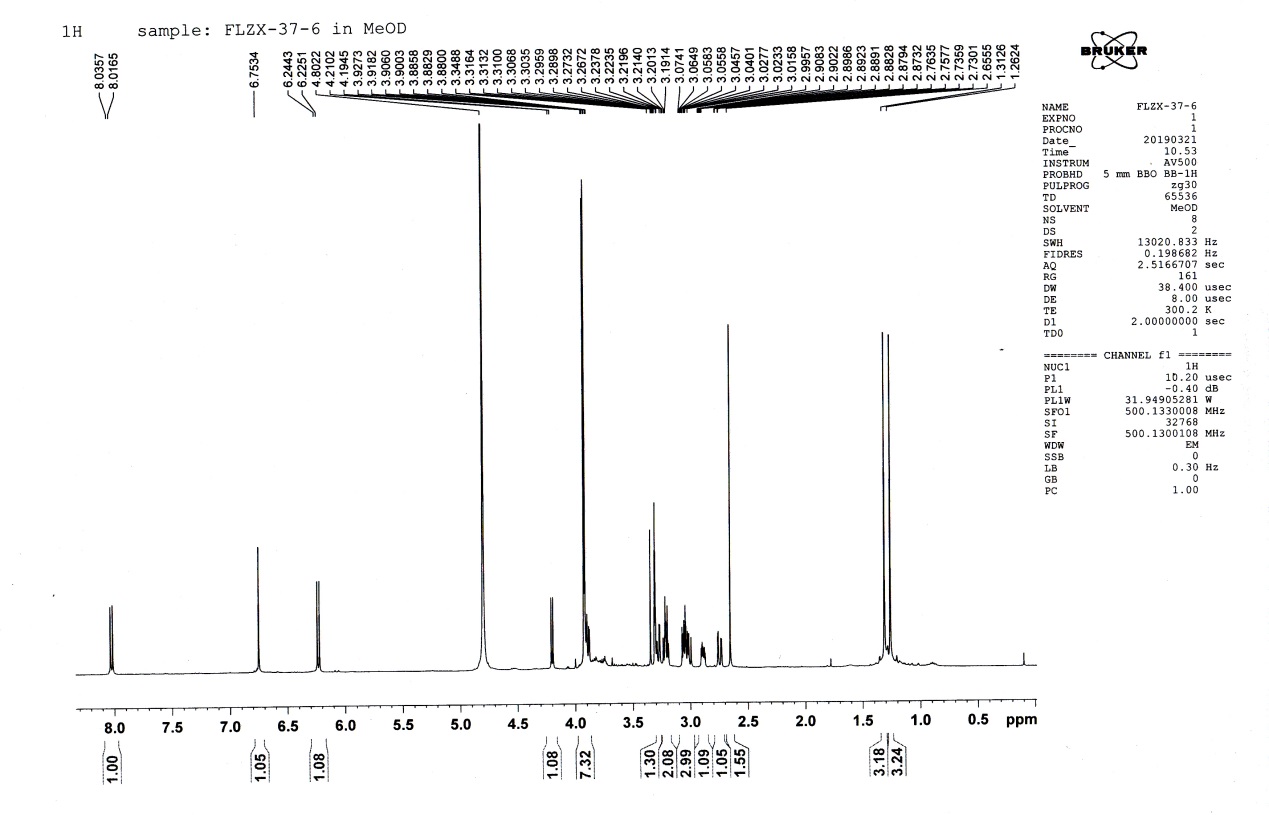


Figure S2. ^1^H NMR spectrum of compound 1


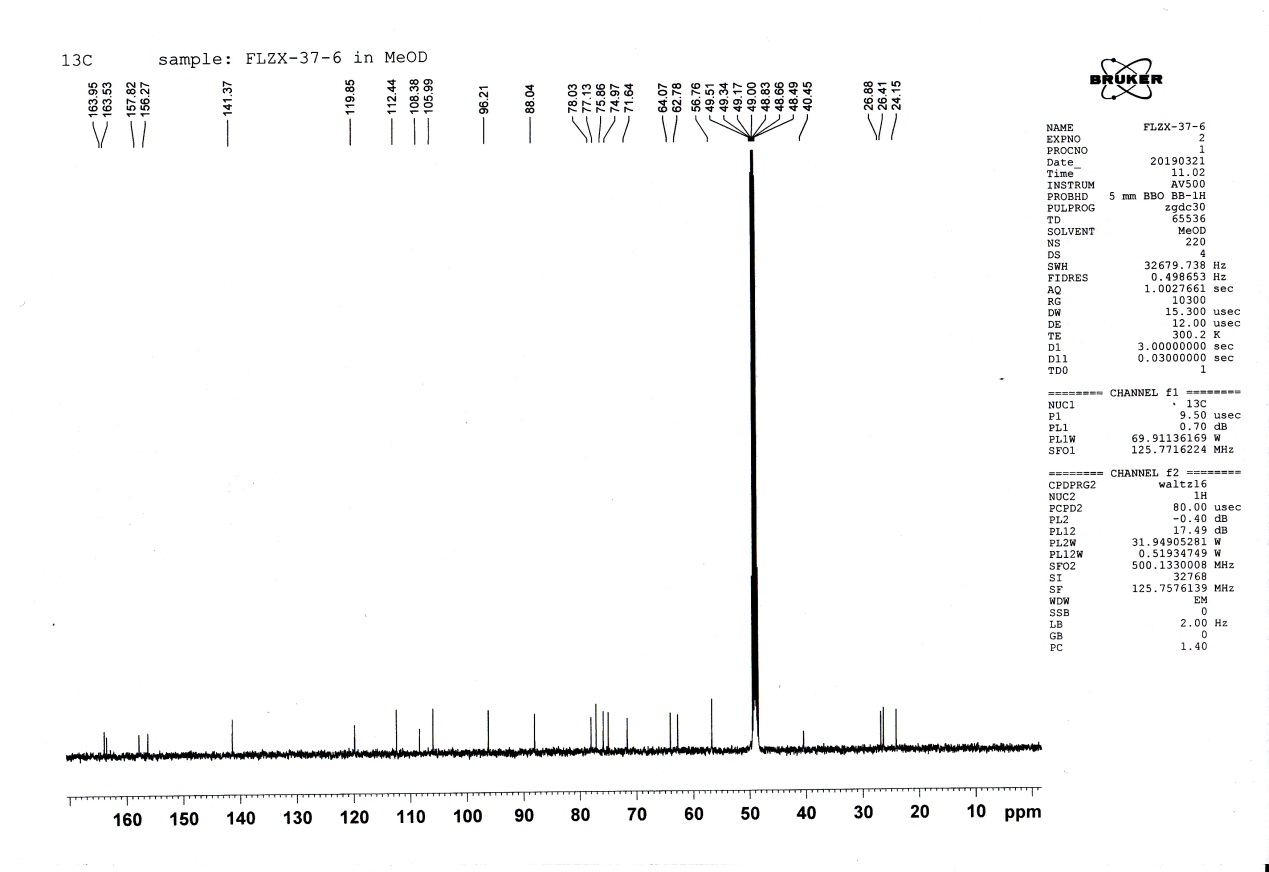


Figure S3. ^13^C NMR spectrum of compound 1


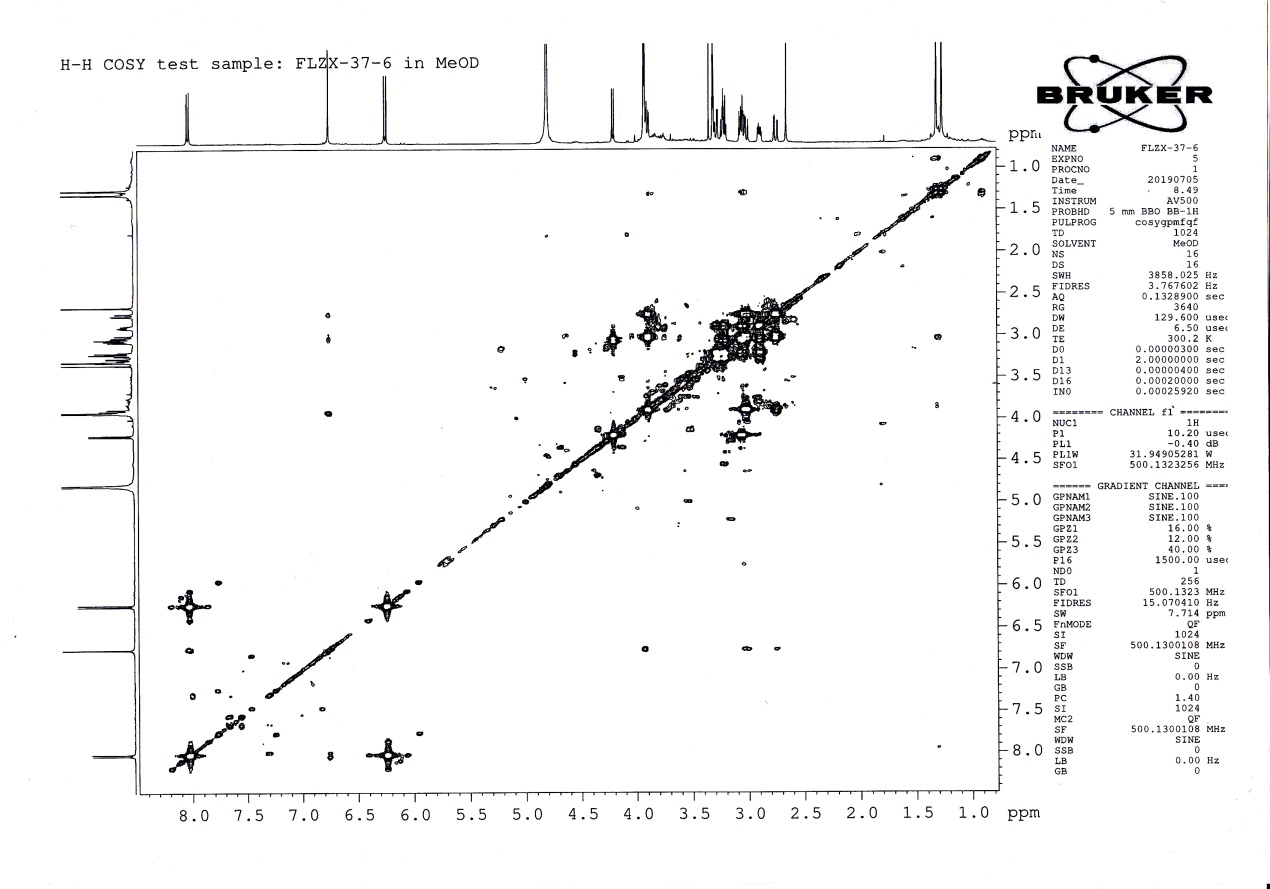


Figure S4. ^1^H-^1^H COSY spectrum of compound 1


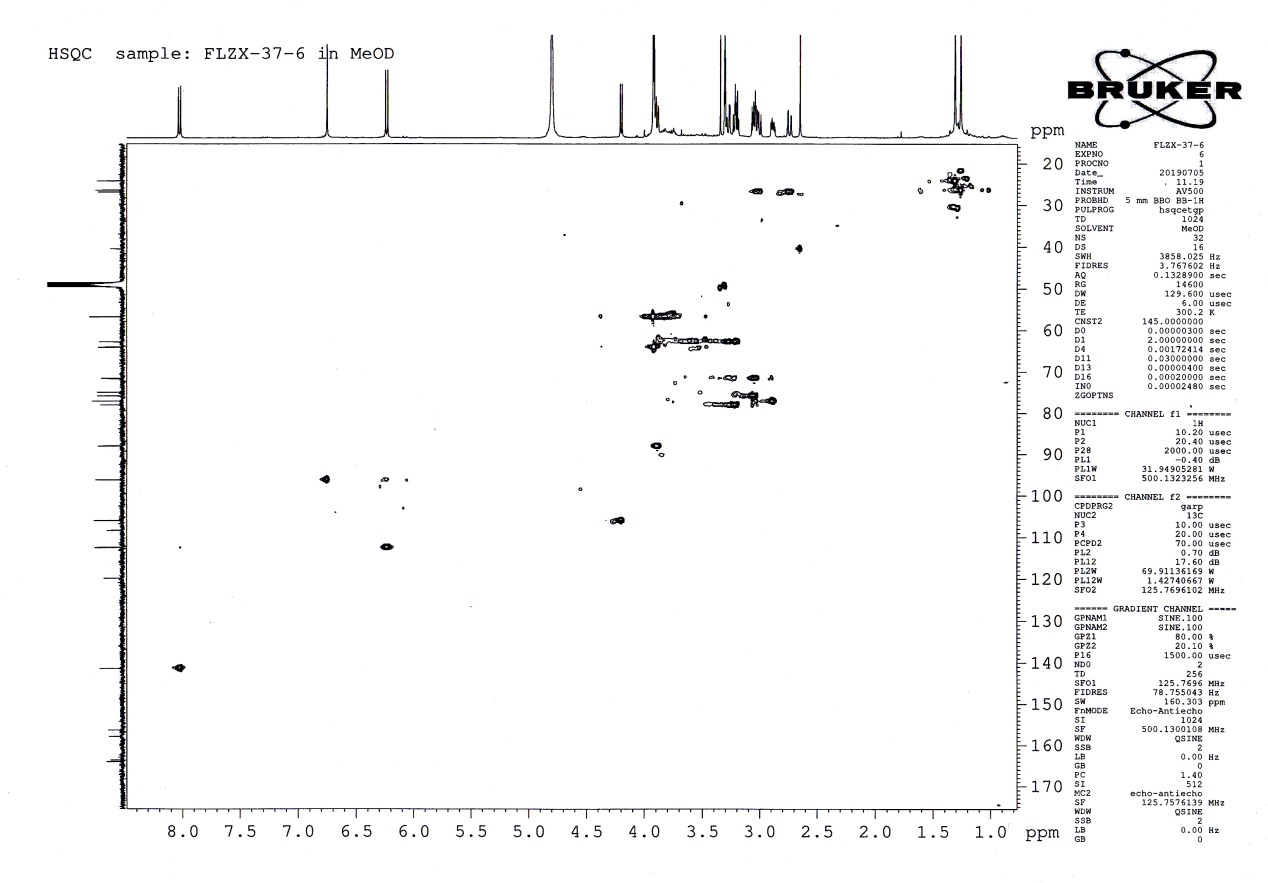


Figure S5. HSQC spectrum of compound 1


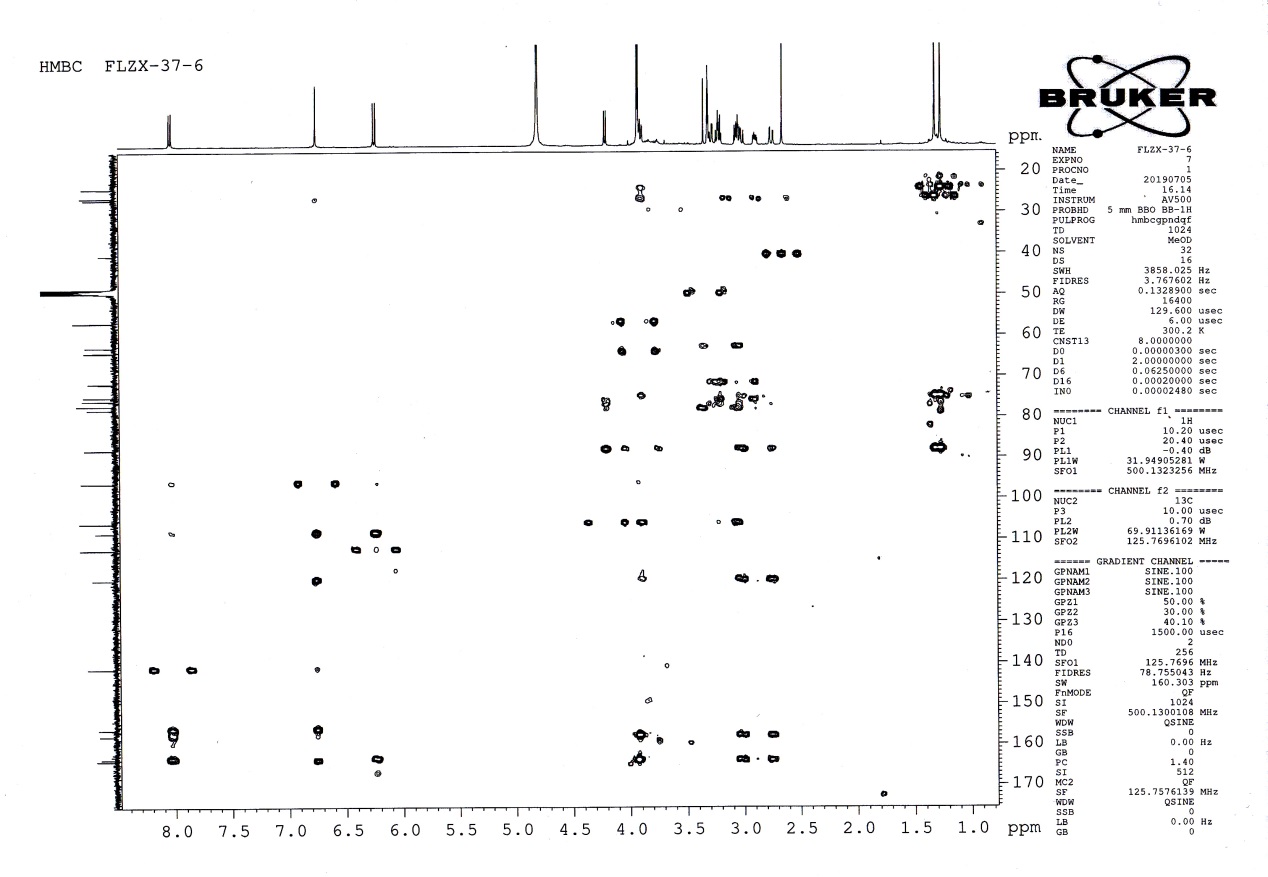


Figure S6. HMBC spectrum of compound 1

Figure S7. HRESIMS spectrum of compound 2


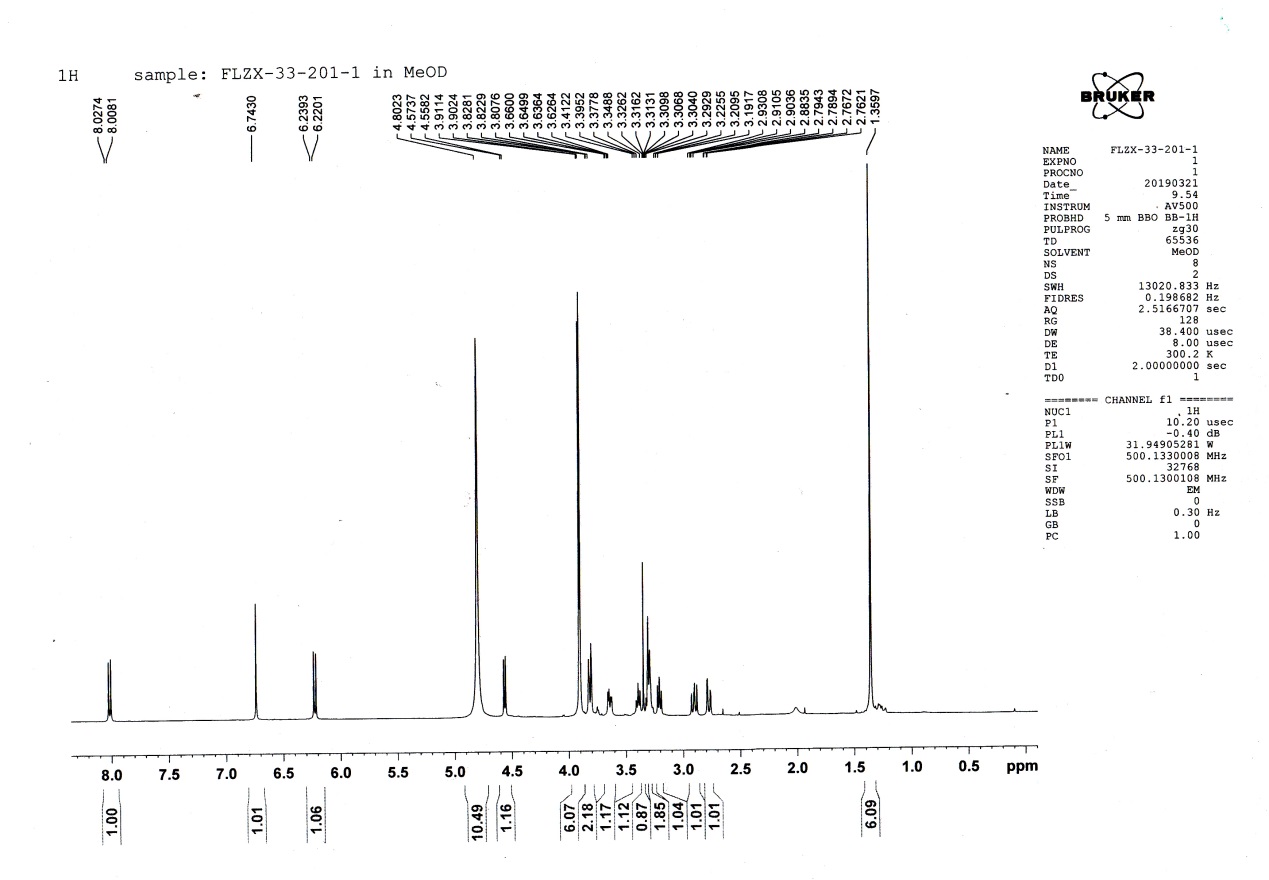


Figure S8. ^1^H NMR spectrum of compound 2


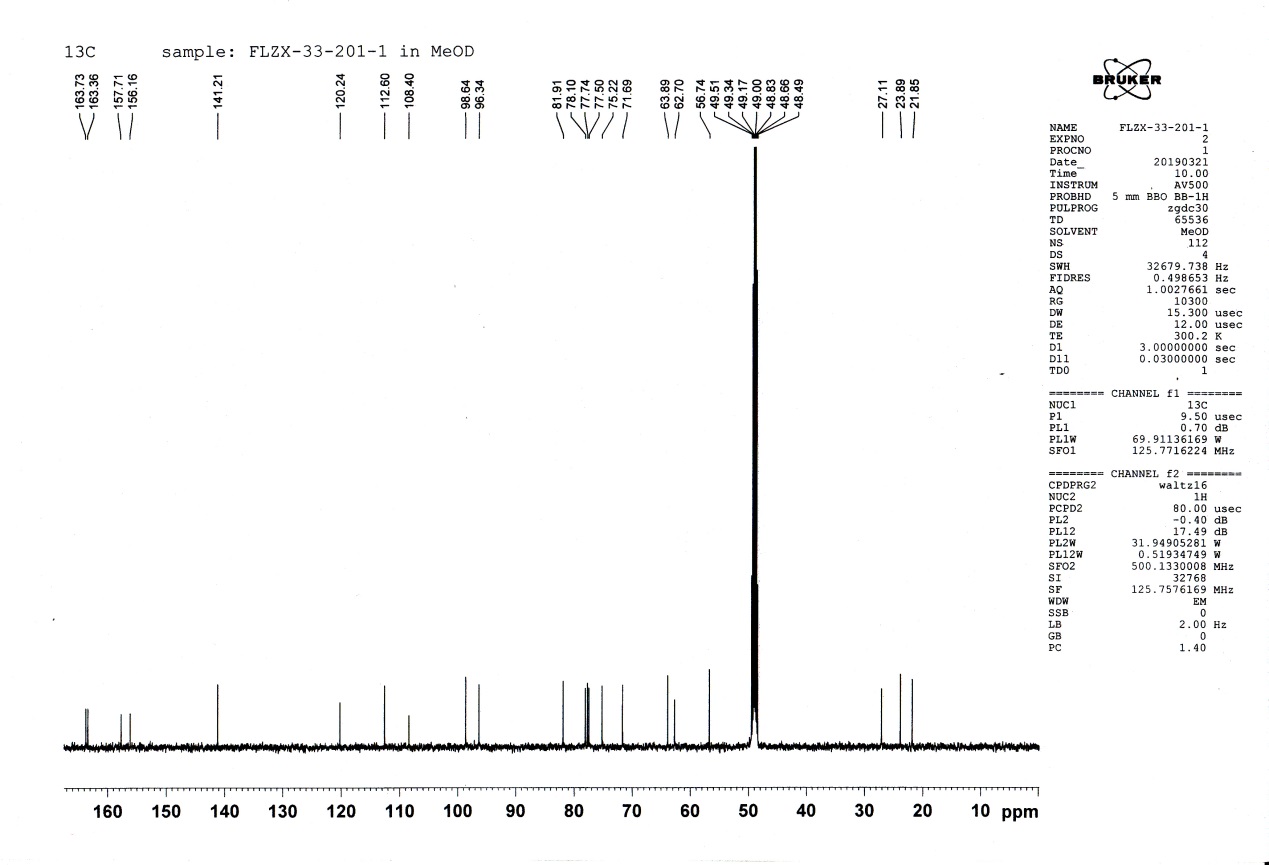


Figure S9. ^13^C NMR spectrum of compound 2


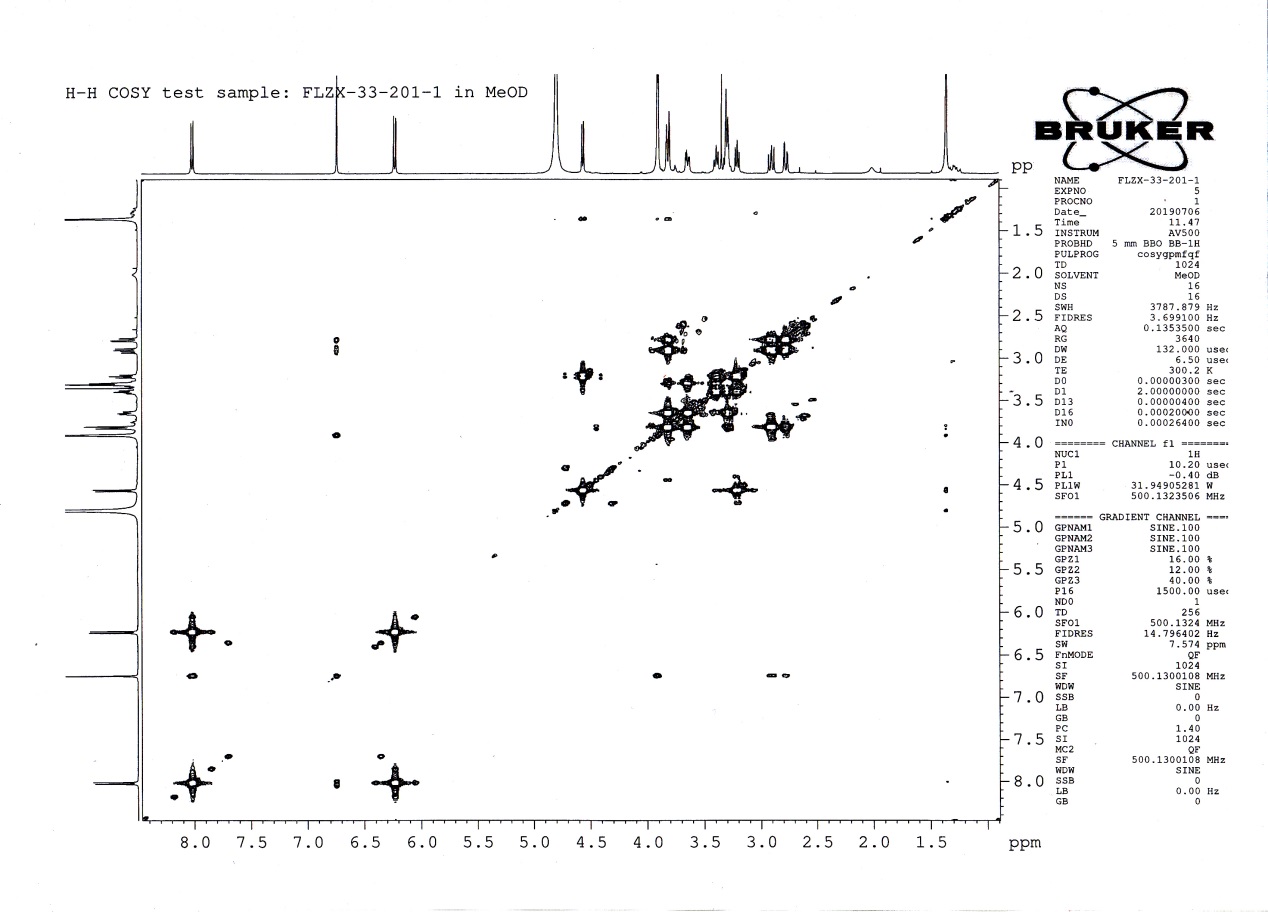


Figure S10. ^1^H-^1^H COSY spectrum of compound 2


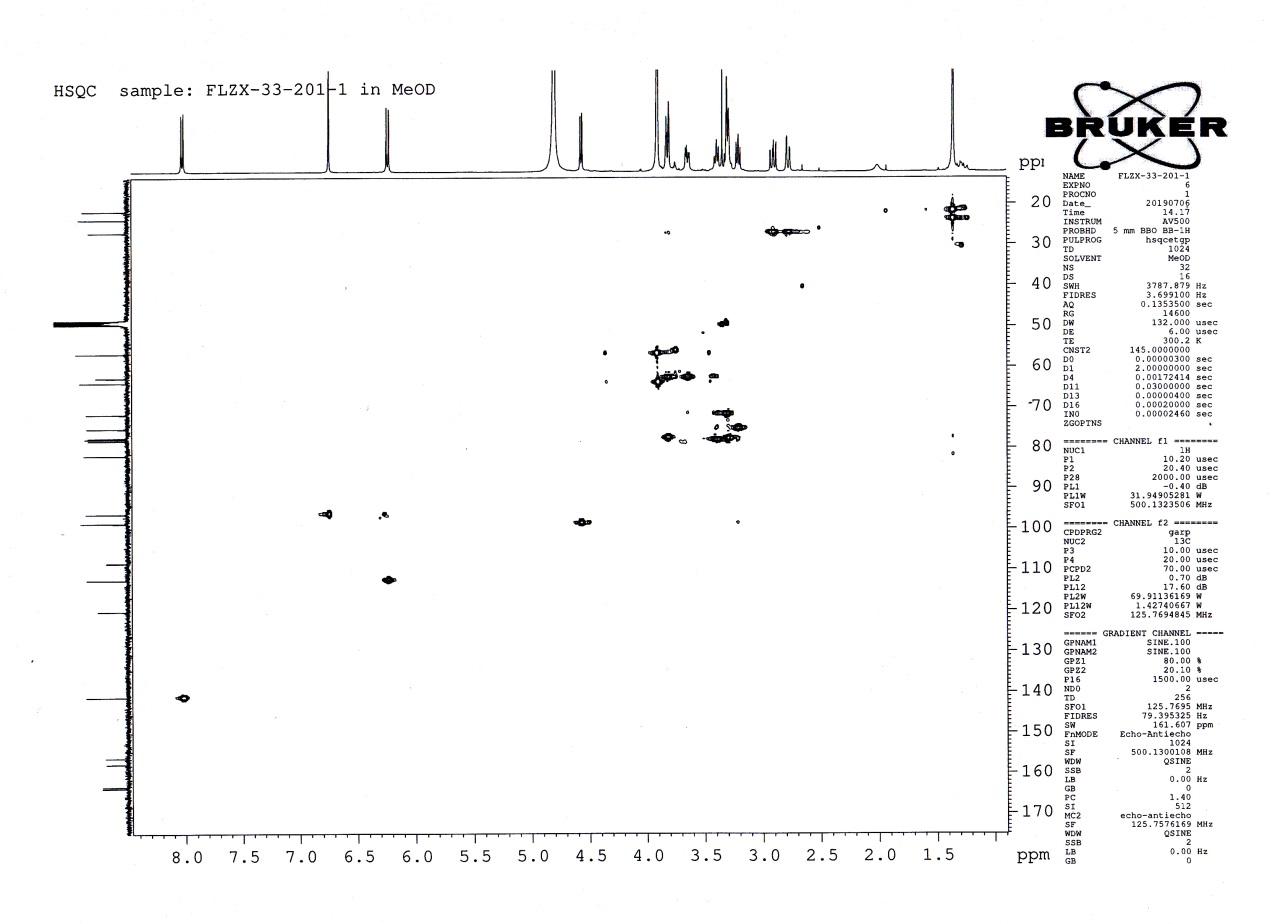


Figure S11. HSQC spectrum of compound 2


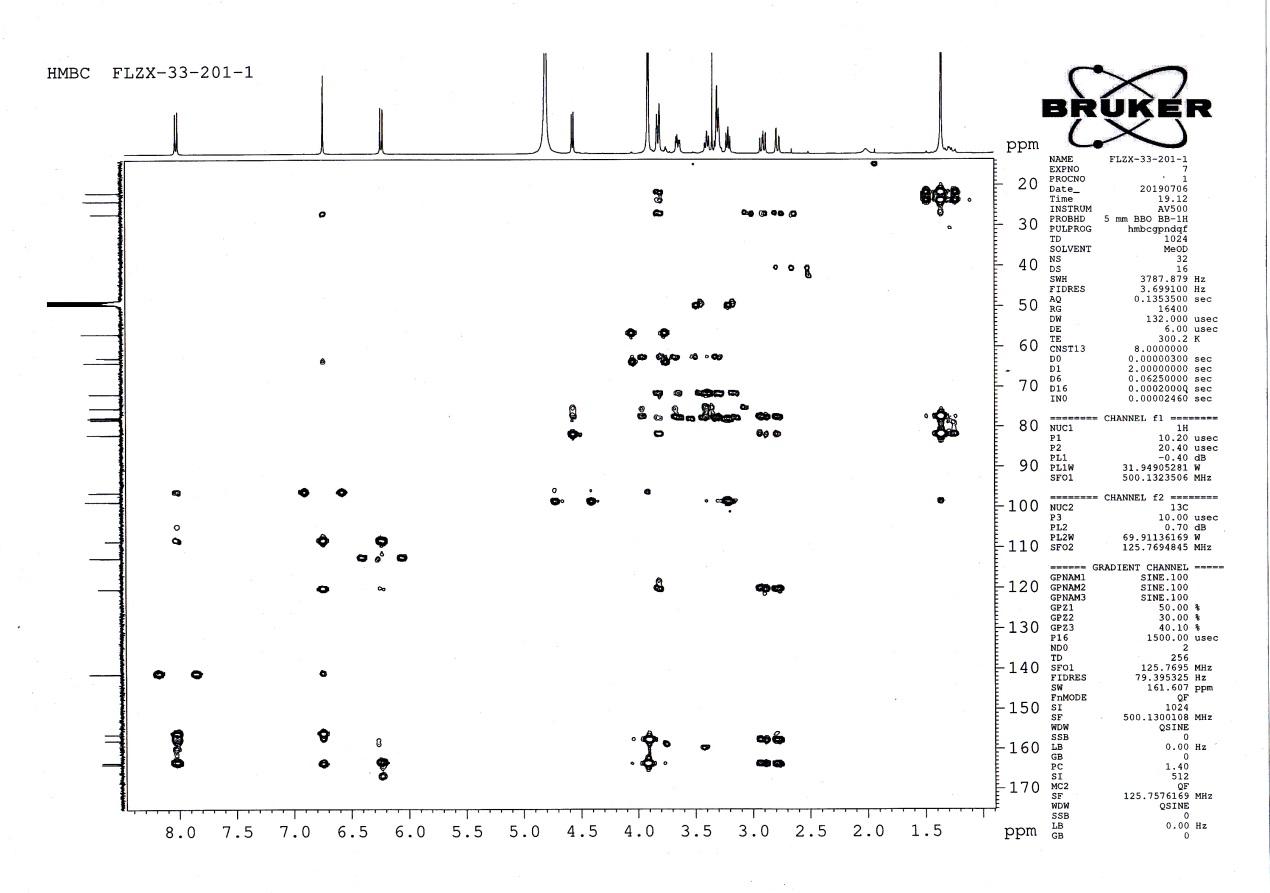


Figure S12. HMBC spectrum of compound 2
